# Insights into the Gut Microbial Communities of Broiler Chicken Fed Black Soldier Fly Larvae-*Desmodium*-Based Meal as a Dietary Protein Source

**DOI:** 10.3390/microorganisms10071351

**Published:** 2022-07-05

**Authors:** Evalyne W. Ndotono, Fathiya M. Khamis, Joel L. Bargul, Chrysantus M. Tanga

**Affiliations:** 1 International Centre of Insect Physiology and Ecology (ICIPE), Nairobi P.O. Box 30772-00100, Kenya; endotono@icipe.org (E.W.N.); jbargul@icipe.org (J.L.B.); ctanga@icipe.org (C.M.T.); 2 Department of Biochemistry, Jomo Kenyatta University of Agriculture and Technology (JKUAT), Kiambu P.O. Box 62000-00200, Kenya

**Keywords:** black soldier fly, gut microbiota, poultry feed, broiler chicken, Oxford nanopore sequencing

## Abstract

The utilization of insect-based diets to improve gastrointestinal function and gut health in poultry is gaining global attention as a promising feed additive. The objective of this study was to determine the optimal inclusion level of the full-fat black soldier fly larvae (BSFL) and *Desmodium intortum* (DI) in broiler chicken diets and to evaluate their impact on the microbial community in the gut. The bacterial communities were characterized using Oxford nanopore sequencing of the full-length bacterial 16S rRNA gene. Four dietary treatments, T1 (25% *DI* + 75% BSFL), T2 (50% *DI* + 50% BSFL), T3 (75% *DI* + 25% BSFL) and T4 (100% fishmeal + 0% *DI* + BSFL), were fed to the broiler chickens for a period of 42 days. Out of the 395,034 classified reads analyzed, the most predominant phyla identified across all the four dietary treatments were *Firmicutes* (94%), *Bacteroidetes* (3%), and *Proteobacteria* (2%). The T1 diet showed the highest alpha diversity and richness according to the Chao1 and Shannon indices. Beta diversity assessment revealed a significant influence of diet on the abundance of the microbiome. There was an increase in beneficial lactic acid bacteria with increasing inclusion of BSFL in the diets. Our findings strongly support the inclusion of BSFL into poultry diet as a promising protein source to reshape the gut microbiota for improved gut health, immune response, and food safety.

## 1. Introduction

Current global population trends have anticipated an increase in the human population to about 9.2 billion people by the year 2050, which will increase the global demand for protein, especially poultry meat [1]. Poultry has the potential to bridge the gap between the demand and supply of protein because of its high feed conversion rates [2]. In the tropics, a large number of smallholder farmers contribute significantly to food security by rearing poultry for domestic and local markets [3]. Broiler chicken meat contributes to the daily dietary needs of humans as it contains protein, minerals, vitamins (niacin, riboflavin, and vitamin B6), and other nutrients [4]. Kenya’s demand and consumption of broiler chicken meat is expected to hit 165,000 metric tons by 2030, up from 55,000 metric tons in 2000 [5]. This projected increase in demand is based on rapid urbanization, middle-class growth, and ever-increasing global health views promoting poultry products as a better protein source than red meat [6]. Protein is an essential key ingredient and one of the most expensive components required in poultry feed. The most common and widely preferred conventional sources of protein in animal feed include soybean, fishmeal, cotton or sunflower seed cakes, and other plant proteins [7]. However, soybean meal and fishmeal are available in low quantities due to land unavailability for production, global cost fluctuation, human consumption of soybean and fishmeal, and other constraints making them much more expensive for farmers to afford [8]. As such, it is important to identify other more affordable, scalable, and sustainable high-quality protein sources to achieve favourable economic returns from animal feed utilization. Insects have been proven to be a good source of feed protein additive over the years [9]. Several insects, including black soldier fly, *Hermetia illucens* L., have been widely promoted as a novel protein source for livestock and fish production because of their positive nutritive properties and year-round availability [10]. Black soldier fly larvae meal can provide high-value feeds that are rich in protein (40–44%) with better amino acid profiles compared to most conventional feeds [11]. The high-quality crude protein found in BSFL could be exploited to produce improved protein-rich animal feeds, thus utilizing BSFL as a better substitute for conventional feeds [8]. The nutritional composition of BSFL, however, depends on the type of organic substrates used for rearing the larvae [12]. Therefore, the integration of BSFL with other important cheap protein sources would be crucial to provide a balanced nutrient composition. Plant-based proteins such as *Desmodium intortum*, which is widely used by smallholder farmers as a fodder crop, have been suggested to be a valuable integration for poultry feed [13]. *Desmodium intortum* (DI) is a large perennial tropical forage legume that grows freely in areas of low temperatures. The most common species used are Greenleaf desmodium and the Silverleaf desmodium [14]. These two species have high-quality protein, can be grown between and under crops, and fixes nitrogen, therefore increasing crop yields and reducing the need for nitrogen fertilizers [15]. In Africa at large, *Desmodium* is being used as an intercrop in a novel cropping system model which is known as push-pull technology, which was developed by the International Centre of Insect Physiology and Ecology (*icipe*) for integrated management of pests such as fall armyworm on cereal crops and has been adopted by over 200,000 smallholder farmers [16]. The cinnamon plant, from the genus *Cinnamomum*, is another plant that has been recently approved as poultry feed additive because it produces bioactive compounds that improve immunity, metabolism, growth performance, and overall poultry health [17]. Previous studies have demonstrated that the intestinal microbiome of poultry is significantly influenced by the dietary ingredients; the nutrient levels of fat, protein, and carbohydrates; the inclusion of feed additives; and dietary supplementation using feed enzymes [18]. The chicken gut microbiota harbours a complex microbial community that plays vital roles in nutrient utilization and adsorption, growth and development, production of short chain fatty acids and overall health status [19]. The microbial diversity is stretched across the entire gut with the main sites being the crop and the ceca [20]. The use of insect-based meal as a suitable alternative source of protein for poultry production is rapidly gaining international research attention, but there is limited information that demonstrates the effects of insect-based meal inclusion in chicken diets and its implications on the intestinal microbiome [21]. There is inadequate knowledge on the potential of black soldier fly larvae meal to transfer pathogenic and beneficial microbes present in the rearing substrates through feed and their impact on the gut health of the birds. Therefore, this study is focused on the assessment of the gut bacterial community dynamics (diversities) and their interactions with the host (broiler chicken) when fed on insect-*Desmodium*-based feeds through metagenomics. This information is crucial and would provide insight on how to improve the practical adoption of safe insect-based products.

## 2. Materials and Methods

### 2.1. Ethical Approval

Ethical approval for this study was provided by the Institutional Animal Care and Use Committee (IACUC) of the Kenya Agricultural and Livestock Research Organization (KALRO)—Veterinary Science Research Institute (VSRI); Approval Code No.: KALRO-VSRI/IACUC019/30082019.

### 2.2. BSFL and Chicken Rearing

The study was mainly based and conducted at *icipe*, Duduville, Kasarani sub-county, Nairobi. The BSFL colony was initiated at the *icipe’s* Animal Rearing and Containment Unit (ARCU) under controlled conditions with a temperature of 28 ± 1 °C and a relative humidity of 70 ± 2%. The 5th instar larvae were harvested from the colony and cleaned by washing in hot water at 84 °C for 10 min. The clean BSFL were oven-dried at 120 °C using a hot air circulating drying oven (Henan Forchen Machinery, Luoyang, Henan, China). The dried BSFL were ground using a Munch hammer mill model M6FFC—230 (Wuppertal, Germany) into powdered larval meal which was used in the formulation of the various test diets. Greenleaf desmodium was harvested from one of the *icipe’s* push-pull plots located at *icipe*, Duduville, and the harvested biomass was dried under a shade and later ground into powder using a milling machine as previously described. At the beginning of the experiment, 120 one-day-old broiler chicks were sourced from Kenchic LTD hatchery in Thika, Kenya and reared at *icipe* for 42 days. The chicks were placed in a brooder room for the first 7 days and the room was maintained at 33–35 °C using light heating bulbs. Before setting up the experiment, all chicks were fed on commercially available starter mash (conventional feed) purchased from Unga Feeds LTD. After the brooding period, the chicks were introduced to defined formulations of BSFL-*Desmodium*-based diets. The chicks were randomly distributed into 12 pens with 10 chicks per pen and each treatment was replicated three times.

### 2.3. Diet Formulations

The test diets were formulated according to the recommendations of the Kenya Bureau of Standards (KEBS) as guided by the NRC (1994) specifications for broiler chicken starter and finisher feeds. The diets were prepared by replacing fishmeal on the control diet with a mixture of BSFL and *Desmodium intortum* as shown in Table 1. All the ingredients required for both the starter diets (Table 2) and finisher diets (Table 3) were sourced and formulated at Unga Limited, Kenya.

### 2.4. Slaughtering of Chickens and Sample Collection

After 42 days, five chickens per pen were randomly selected for sampling. The birds were sacrificed humanely by a professional expert following the IACUC-approved protocols. The gut contents were harvested, and samples were excised from the following eight major regions: esophagus, crop, proventriculus, gizzard, duodenum, small intestines, large intestines, and cecum. The samples were placed in sterile Eppendorf tubes and kept at −20 °C at the Arthropod Pathology Unit (*icipe*, Kenya) to await further analysis.

### 2.5. Genomic DNA Extraction and 16S rRNA Amplification

Gut contents and inner epithelial tissues were collected for genomic DNA isolation using the Isolate II Genomic Extraction kit (Bioline, London, UK) following the manufacturer’s instructions. The concentration and quality of DNA was determined using a Nanodrop 2000/2000 c spectrophotometer (Thermo Fischer Scientific, Wilmington, MA, USA) and good quality DNA samples with an absorbance range of A_260nm_/A_280nm_ 1.8–2.0 were selected for metagenomics processing. The full-length bacterial 16S rRNA gene of ~1500 bp was sequenced using Oxford Nanopore Technologies (ONT) Minion device with R9.4.1 flow cells and the libraries were prepared with pooled DNA for multiplexing using the 16S barcoding kit SQK-16S024. Library preparation with a PCR step was conducted using the following components: 10 pmol μL^−1^ of each 16S barcode, 10 ng μL^−1^ of DNA template, 0.625 U μL^−1^ *MyTaq* DNA polymerase (Bioline), and 5X *MyTaq* reaction buffer (5 mM dNTPs, 15 mM MgCl_2_, stabilizer, and enhancer) (Bioline). The reactions were set up in a total reaction volume of 50 µL and run in a Mastercycler Nexus gradient thermal cycler (Eppendorf, Germany), under the following conditions: initial denaturation for 2 min at 95 °C, followed by 35 cycles of denaturation for 30 s at 95 °C, annealing for 40 s at 55 °C, extension for 1 min at 72 °C, and a final extension step of 10 min at 72 °C. The libraries were then purified using a Bioline kit according to the manufacturer’s instructions and pooled together before loading into the flow cells for Minion sequencing.

### 2.6. Sequencing and Data Analysis

The sequencing was done for 4 h with live base calling being performed using the Albacore tool (v 2.3.4) on the Minknow (v 20.10.3) software on the ONT cloud. The reads that passed sequencing generated FASTQ files which were uploaded to EPI2ME software v 2020.11.19 (https://epi2me.nanoporetech.com (accessed on 12 November 2021)) for qualitative, real-time species identification from metagenomic samples. The FASTQ-16S workflow (v 2020.04.06) on EPI2ME was used to characterize the reads and assign taxonomy to the genus level using NCBI as the reference database and relative cumulative abundance plots were generated. A minimum abundance cut-off of 0.1% was used to select the most abundant taxa and those that were below were collapsed into others. However, EPI2ME is a limited tool since it does not give the diversity parameters such as alpha and beta diversity. The q2ONT pipeline (https://github.com/DeniRibicic/q2ONT (accessed on 5 December 2021)), which uses features and tools embedded in QIIM2 v 2020.8 [22] was used for further processing to generate the reads needed for bacterial diversity statistics. On the QIIME2 pipeline, the adapters were trimmed using the trimmomatic tool (v 0.39) and the reads were demultiplexed using the ONT porechop tool (v 0.2.4). The demultiplexed reads were checked for chimeric sequences using the VSEARCH Qiime2 tool and the chimeric sequences found were filtered out using the UCHIIME tool. The reads were then aligned to MAFFT and taxonomy was assigned using a pre-trained SILVA 138 database as the reference database. Operational Taxonomic Units were generated from the reads using an 85% similarity threshold. The reads were then rarefied to even sampling depths and both alpha and beta diversities were calculated. Alpha diversity was calculated using the Shannon index and Chao1 index using the phyloseq package [23] in R. To compute the microbial beta diversity, dissimilarity analyses were performed and sample Bray–Curtis distances were visualized on PCoA plots. Multivariate analysis of beta diversity was verified using non-parametric PERMANOVA using the Adonis function in R with 999 permutations (*p <* 0.05).

## 3. Results

**Figure 1:**
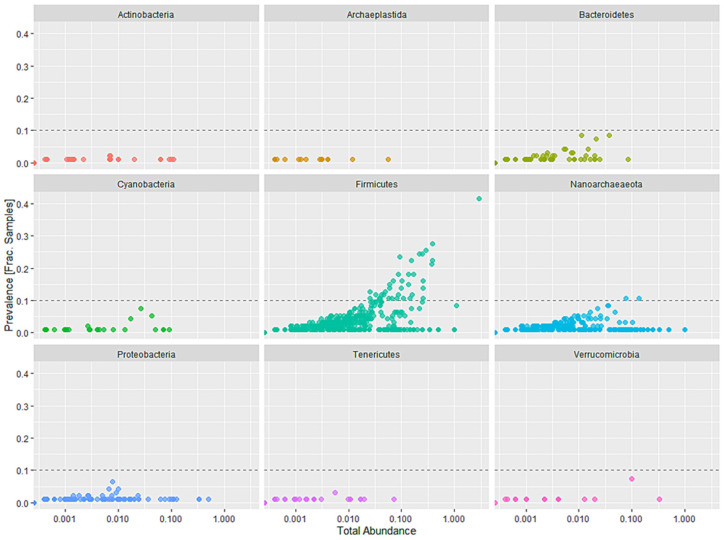
Prevalence of the most dominant phyla as observed across all the four dietary treatments. Prevalence shown is in relation to the total abundance counts.

**Figure 2:**
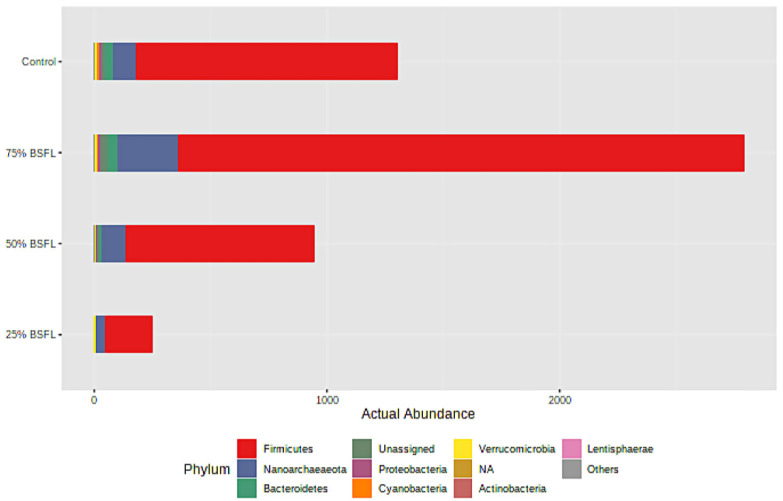
Cumulative relative abundance of the predominant phyla groups observed across all the four dietary treatments.

Minion nanopore sequencing obtained a total of 395,034 raw reads from which 292,100 reads passed the quality filtering. Out of these reads, T1 had 100,912 reads, T2 had 72,404 reads, T3 had 38,500, and T4 (control) had 80,309 reads. All samples were rarefied to even sampling depths with an average of 20,000 reads/sample and 984 OTUs were identified after quality sampling. The most abundant bacterial phyla observed across all the dietary treatments were *Firmicutes* (94%), *Bacteroidetes* (3%), and *Proteobacteria* (2%), with lesser amounts of *Verrucomicrobia*, *Actinobacteria*, *Cyanobacteria*, and *Tenericutes* (Figure 1). The phylum *Firmicutes* significantly increased in T1 as compared to T4 (Figure 2).

**Figure 3:**
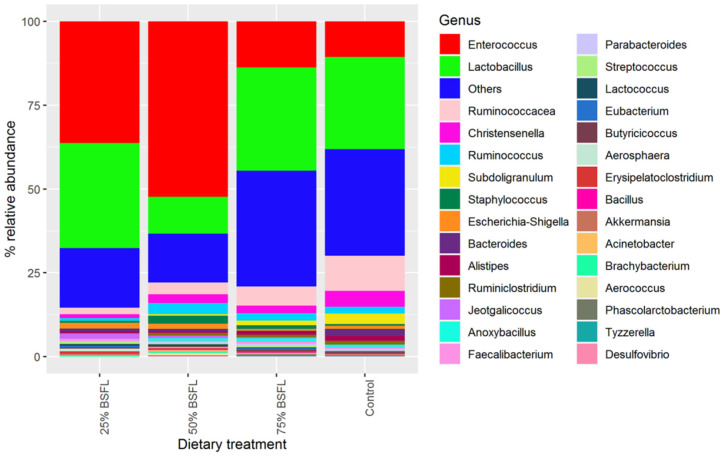
Cumulative relative composition of bacteria operational taxonomic units at genus level observed across the different dietary treatments. The top 30 genera with high abundance were selected to calculate the relative abundance.

The OTUs clustering in the families of *Enterococcaceae*, *Lactobacillaceae*, *Ruminococcaceae*, and *Lachnospiraceae* were predominant in most of the samples, with the family *Enterococcaceae* being the most abundant (Figure S1). At the genus level, the cumulative relative abundance across the different diets showed *Enterococcus*, *Lactobacillus*, and *Ruminococcus* to be the most predominant genera (Figure 3). The genus *Enterococcus* increased in diets that had BSFL inclusion than the control diet and was most abundant in the diet that had T2 while the genus *Ruminococcus* was higher in the control diet than in the rest of the treatments (Figure 3).

**Figure 4:**
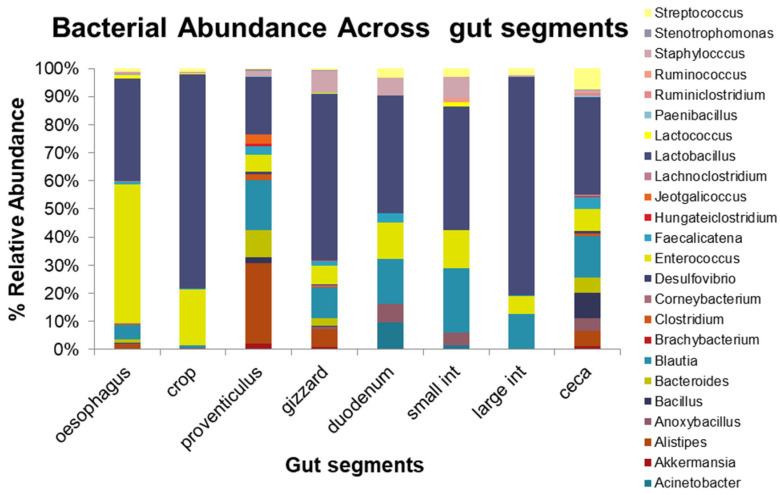
Stacked bar graph showing the relative abundance of the gut bacterial communities across the different gut segments.

*Lactobacillus* increased significantly in T1 compared to T4. Across all the gut segments sampled, the genus *Lactobacillus*, *Enterococcus*, *Blautia,* and *Alistipes* dominated along this part of the guts. *Lactobacillus* was dominant in the crop, gizzard, duodenum, small intestines, large intestines, and cecum. *Enterococcus* was dominant in the oesophagus while *Alistipes* was dominant in the proventriculus (Figure 4). The highest microbial diversity was observed in the ceca while the lowest diversity was in the duodenum, small intestines, and large intestines.

**Figure 5:**
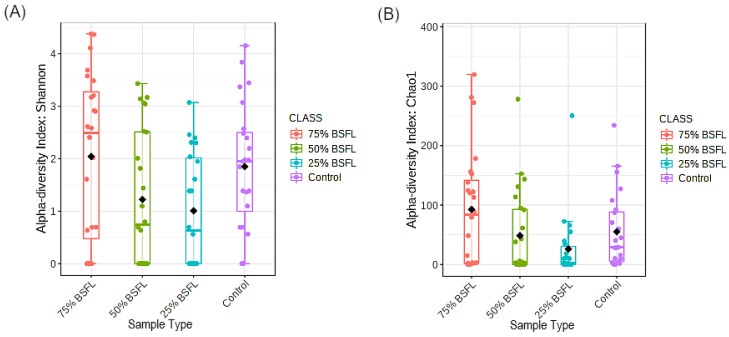
Alpha diversity estimate measures by (**A**) Shannon index and (**B**) Chao1 index from the gut bacterial profiles of our samples.

Alpha diversities were assessed by the Shannon and Chao1 indices and the diet with 75% BSFL (T1) inclusion recorded the highest diversity according to the Shannon index (Figure 5A) and the highest bacterial OTU richness according to Chao1 index (Figure 5B). The diet treatment with 25% BSFL (T3) inclusion had the lowest diversity. However, this difference was not statistically significant (*p*-value > 0.05).

**Figure 6:**
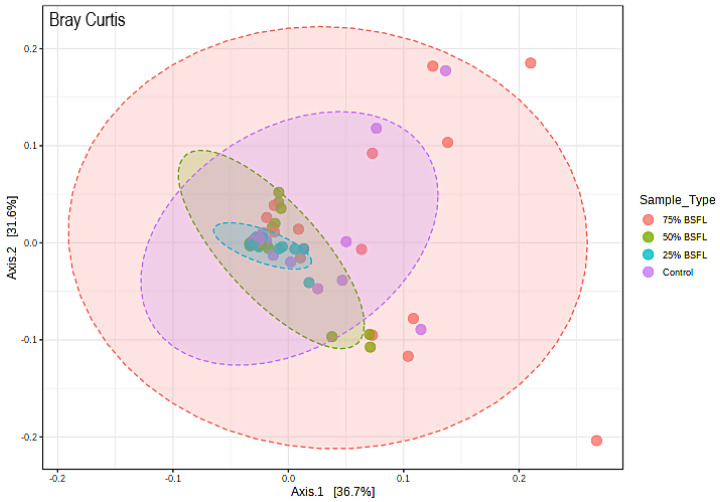
Beta diversity PCoA plot based on Bray-Curtis distance dissimilarity method between the different dietary treatments. PERMANOVA R-Squared: 0.049; *p*-value > 0.001.

Beta diversity calculations showed that very few changes were observed in the abundance of microbial communities present in the samples. All samples shared almost similar communities regardless of diet treatment. When phylogenetic distances were considered via Bray Curtis (Figure 6), the bacterial communities clustered together regardless of diet treatment. This similarity was confirmed by PERMANOVA (*p*-value *>* 0.05).

**Figure 7:**
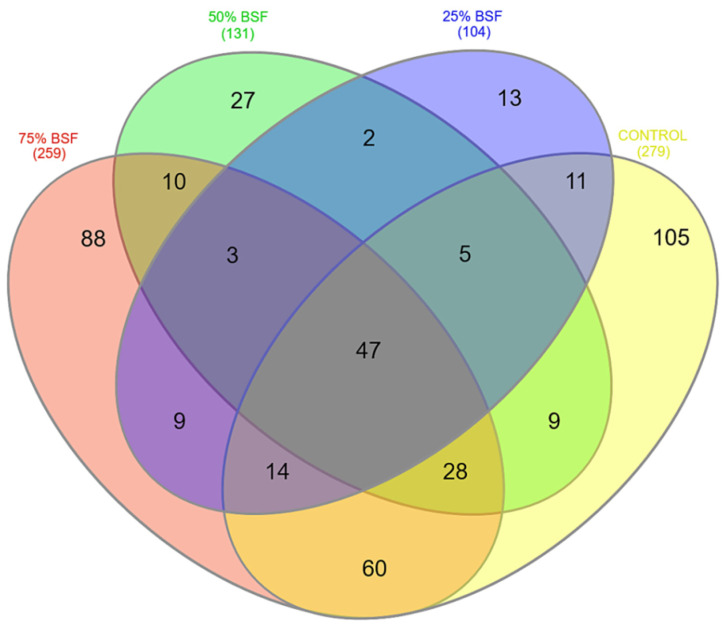
Venn diagram showing the unique and shared bacteria genera between the four dietary treatments.

A Venn diagram showed that 47 bacteria genera were common across all the four treatments (Figure 7). The control (T4) treatment had the most unique genera, with a total of 105, followed by the 75% BSFL treatment (T1) with 88 unique genera. Most of the OTUs were shared between the control diet (T4) and the 75% BSFL (T1) diet.

## 4. Discussion

The use of low-cost insect-based feed as an alternative to the expensive conventional fish/soya bean meal based has gained global research attention recently to improve the sustainability of poultry production, which will in turn feed the growing human population [24,25]. Here, we present the first report that evaluates the effects of combining BSFL and *Desmodium* into the feed ratio on the gut bacteria community shift when compared to those fed conventional feeds using Oxford nanopore sequencing technology. According to our study’s findings, a BSFL-based diet can replace fishmeal in broiler chicken diets without negatively impacting their gut microbiome and overall health status. A study reported by [6] on the growth performance of broiler chickens fed BSFL-*Desmodium* feed showed that during the initial growth stages, there were significant effects of fishmeal replacement with BSFL-based feed on the average weight gain, daily body weight gain, and average food intake. Similarly, findings reported previously [26] showed dietary inclusion of BSFL had a positive influence on the cecal microbiota and mucin composition in broilers supports our results that BSFL diets have a positive impact on the gut microbiome of broilers. *Firmicutes*, *Bacteroidetes*, and *Proteobacteria* were detected to be the most abundant bacterial phyla across all diets, with lesser portions of *Actinobacteria*, *Tenericutes*, *Verrucomicrobia*, and *Cyanobacteria*. In our present study, the *Firmicutes* were predominant over the *Bacteroidetes* and *Proteobacteria*; this is in agreement with other previous reports [27,28]. The gut community was colonized by bacteria families of *Enterococcaceae*, *Lactobacillaceae*, *Ruminococcaceae*, and *Lachnospiraceae*. These results are comparable to other reported studies on the intestinal microbiome of broiler chicken [29].

The phylum *Firmicutes* contains the majority of the beneficial bacteria that are useful to both animals and humans. *Firmicutes* play a key role in the relationship between the intestinal bacteria and chicken health. Several members of this phylum are used as probiotics including *Lactobacillus*, *Enterococcus*, and *Faecalibacterium* [30]. The predominant bacteria genera observed across all treatments in our study were *Lactobacillus* and *Enterococcus*. The diets with BSFL-*Desmodium* meal inclusion supported the same microbial communities as the standard fishmeal diet at both the phylum and genus levels with only a few taxa changing in abundance. This suggests that our diet is a good replacement for conventional fishmeal, since no significant changes were observed in microbial communities. Across the gut segments, the highest microbial diversity was observed in the distal part at the caecum while the lowest diversity was in the duodenum, small intestines, and large intestines. The microbial composition density in the caecum may have increased due to the long feed retention time, availability of nutrients to digest, and the weakening of antibacterial potency of digestion fluids [27]. On the other hand, the proximal parts had low bacteria abundance which may have been affected by factors such as low pH, which limits growth of many bacteria species, rapid passage of the digesta especially in the ileum, and availability of bile salts in the ileum, which may reduce the proliferation of many bacteria species [31]. The proximal parts were mainly dominated by the *Lactobacillus* bacteria and lesser portions of *Enterococcus* while the distal part (caecum) was dominated by *Lactobacillus*, *Blautia*, *Bacillus*, *Enterococcus,* and *Alistipes*. Our findings are in agreement with the previous reports which found that *Lactobacillus* dominated across the gut sections whereas the caecum is dominated by bacteria from *Firmicutes*, *Bacteroidetes*, and *Proteobacteria* [18,28]. A significant increase in the lactic acid bacteria (LAB) such as *Lactobacillus* and *Enterococcus* was observed in the diets that had BSFL inclusion, especially in the diet with 75% BSFL. This positive increase in LABs, which are also short-chain fatty acid (SCFA)-producing bacteria, could be related to the presence of chitin in the BSFL diet. As suggested in other studies [32], chitin is a naturally occurring polysaccharide that can be fermented by microbes, degraded by SCFA-producing bacteria, and may also serve as a substrate for microbes. The lactic acid produced by LABs could facilitate fermentation that produces hydrolytic enzyme that acts on proteins and polysaccharides such as chitin [33]. Studies have reported that high bacterial diversity is regarded to be beneficial to gut health because rich communities are likely to compete with pathogens for resources and colonization, preventing pathogen invasion and infection [34]. Therefore, the broiler chickens in the study may have a healthier gut community, since they had higher microbial diversity than those fed a conventional fishmeal diet. LABs are innocuous microorganisms that can produce bacteriocins, organic acids like lactic acid, hydrogen peroxide, diacetyl, and carbon dioxide, among other inhibitory agents. Lactic acid bacteria can counter pathogenic microbes through their competitive exclusion mechanism based on competition for binding sites and nutrients, prevention of the pathogens’ adhesion, modulation of the host immune system, and reducing the bioavailability of toxins [35]. In animal production, LABs have been known to reduce the risk of infections and intestinal disorders associated with pathogens [35]. Therefore, LABs have several advantages as potential probiotics and can be used in place of antibiotics in poultry production. Bacteria genera belonging to *Lactobacillus*, *Enterococcus*, *Bacillus*, *Bifidobacterium*, *Streptococcus*, and *Faecalibacterium* are some of the probiotic species commonly being used and they have been identified to have a beneficial effect on broiler nutrition. These desirable benefits include broiler chicken growth performance, modulation of gut microbial communities, pathogen inhibition, immunomodulation, improved meat sensory features, and enhancing broiler chicken meat’s microbiological quality [36]. These beneficial effects on broiler chicken’s nutrition may have a positive impact on human nutrition, since there would be a low risk of contamination and spread of diseases, such as campylobacteriosis and salmonellosis. *Lactobacillus*, which was prevalent across all diets in this study, remains the probiotic that has received the most attention in poultry production and animal research. This bacteria genus is predominant in the chicken’s gastrointestinal tract and plays an important role in chicken physiology by producing lactic and acetic acids that lower pH in the gut, competes for nutrients and adhesion sites with enteropathogenic bacteria such *Campylobacter*, *Salmonella*, and *Escherichia coli* [37], and has excellent tolerance properties to acids and bile salts [38]. Previous studies have reported the potential of *Lactobacillus* to have anti-*Campylobacter* activity against the zoonotic disease campylobacteriosis found in contaminated poultry meat [39]. As suggested by [17], dietary cinnamon inclusion in poultry feed could also increase the growth of *Lactobacillus* while inhibiting *Campylobacter* in the ileum and caecum of poultry. In addition, *Lactobacillus* has also been shown to have antimicrobial activity against *Salmonella* in chickens [37]. In pigs production, *Lactobacillus* spp. has been reported to promote improved performance, improved immune systems, and control of post-weaning diarrhoea, among other benefits [40]. In human health, *Lactobacillus* spp. has been reported to have a variety of health benefits, including immune stimulation, anti-cancer activity, prevention and treatment of inflammatory diseases [41], lactose intolerance relief, and antimicrobial activity against resistant pathogens and respiratory viral infections [42].

In this study, we found that the *Enterococcus* genus was more abundant in diets that contained BSFL compared to the control group consisting of 100% fishmeal diet. The genus *Enterococcus*, belonging to the family *Enterococcaceae* could work as a probiotic albeit to a much lesser extent than *Lactobacillus*. Unlike the *Lactobacillus* probiotic strains, reports on the effectiveness of *Enterococcus* strains as probiotics remain limited. Established probiotic species from this genus include *E*. *faecalis* and *E. faecium* [43]. In humans, *E. faecium* has been shown to inhibit the growth of pathogens such as *E. coli*, *Salmonella*, *Shigella*, and *Enterobacter*, making the strain suitable for use as a treatment for diarrhoea, irritable bowel syndrome, immune regulation, and lowering serum cholesterol [44]. In poultry research, studies have shown that *Enterococcus* as a probiotic has a positive impact on poultry health [31,45]. In a research study done by [46], the effect of *E. faecium* on the growth performance of broiler chickens was investigated, and they observed that probiotic treatment considerably improved broiler chick performance in terms of weight gain and feed conversion ratio. Another study on *E. faecium* strain on broiler chickens observed that the bacterium significantly increased the antioxidant status, bilirubin content, serum calcium content, and body mass of the broiler chickens [47]. Other potential probiotic bacteria were also identified in our study, including *Faecalibacterium*, *Bacillus*, and *Streptococcus*. All these LABs are being exploited extensively and are being considered as a safe alternative to growth promoters and antibiotics and as feed supplements to improve growth performance [18]. This may indeed lead to a shift away from the use of antibiotics in poultry production.

In conclusion, our research study was successful in identifying bacterial taxa that are beneficial to poultry health. These beneficial taxa could be further exploited in the poultry industry to produce probiotics and prebiotics to improve poultry health and nutrition. BSFL-*Desmodium* inclusion in the broiler diet has shown to be a promising feed additive as a replacement for fishmeal and demonstrated the ability to enhance gut health and modulate gut microbial diversity by increasing the abundance of lactic acid bacteria in broiler chicken. Therefore, the use of insect and legume-based protein mixture may be a potential and promising protein-rich additive in broiler chicken diets and insect-based feed technologies can be promoted alongside push-pull technologies that involve the widespread use of *Desmodium* by over 200,000 smallholder farmers [48].

## Figures and Tables

**Table 1:** Summary of diet formulations into four treatments using combinations of varying proportions of BSFL (Black Soldier Fly larvae) and *Desmodium intortum* in place of the conventional fishmeal diet.

Treatment Group, T	Diet Formulation
T1 (Treatment 1)	25% *Desmodium intortum* + 75% BSFL
T2 (Treatment 2)	50% *D. intortum* + 50% BSFL
T3 (Treatment 3)	75% *D. intortum* + 25% BSFL
T4 (Control)	100% Commercial fishmeal

**Table 2:** Feed composition for broiler chicken starter (Diets (g/kg) as fed) of experimental diets.

Ingredients (%)	Control	T1	T2	T3
Maize germ	528.8	527.0	540.0	550.3
Wheat pollard	104.0	108.0	97.9	201.6
Corn oil	24.6	16.3	11.4	4.0
Fish meal	16.3	0.0	0.0	0.0
*Desmodium intortum*	0.0	82.7	165.3	247.0
BSFL	0.0	247.9	165.3	82.7
Limestone	10.0	10.0	10.0	10.0
Salt	3.6	3.6	3.6	3.6
Di-calcium phosphate	0.5	1.0	3.3	3.2
Broiler premix ^1^	2.5	2.5	2.5	2.5
Mycotoxin binder	1.0	1.0	1.0	1.0

^1^ Broiler premix (provided per kg of diet) = vitamin A, 6,250,000 IU; vitamin D3, 1,000,000 IU; vitamin E, 15,000 IU; vitamin K3, 1000 mg; vitamin B1, 500 mg; vitamin B2, 2500 mg; vitamin B6, 2500 mg; vitamin B12, 10 mg; pantothenic acid, 600 mg; nicotinic acid, 15,000 mg; folic acid, 500 mg; biotin, 35 mg; choline chloride, 150,000 mg; iron, 20,000 mg; copper, 2500 mg; zinc, 25,000 mg; manganese, 15,000 mg; iodine, 600 mg; cobalt, 400 mg; BHT (anti-oxidant), 125,000 mg. T1 = 2 5% *D. intortum* + 75% BSFL, T2 = 50% *D. intortum* + 50% BSFL and T3 = 75% *D. intortum* + 25% BSFL and Control = commercial feed.

**Table 3:** Feed composition for Broiler starter finisher diets (Diets (g/kg) as fed) of Experimental Diets.

Ingredients (%)	Control	T1	T2	T3
Maize germ	550.0	526.0	534.0	576.5
Wheat pollard	201.6	200.5	198.2	165.5
Corn oil	27.2	22.1	15.2	5.4
Fish meal	191	0.0	0.0	0.0
*Desmodium intortum*	0.0	55.0	110.0	165.0
BSFL	0.0	165.0	110.0	55.0
Limestone	22.6	22.6	22.6	22.6
Salt	3.6	3.6	3.6	3.6
Di-calcium phosphate	0.5	1.7	2.9	3.3
Broiler premix ^1^	2.5	2.5	2.5	2.5
Mycotoxin binder	1.0	1.0	1.0	1.0

^1^ Broiler premix (provided per kg of diet) = vitamin A, 6,250,000 IU; vitamin D3, 1,000,000 IU; vitamin E, 15,000 IU; vitamin K3, 1000 mg; vitamin B1, 500 mg; vitamin B2, 2500 mg; vitamin B6, 2500 mg; vitamin B12, 10 mg; pantothenic acid, 600 mg; nicotinic acid, 15,000 mg; folic acid, 500 mg; biotin, 35 mg; choline chloride, 150,000 mg; iron, 20,000 mg; copper, 2500 mg; zinc, 25,000 mg; manganese, 15,000 mg; iodine, 600 mg; cobalt, 400 mg; BHT (anti-oxidant), 125,000 mg. T1 = 2 5% *D. intortum* + 75% BSFL, T2 = 50% *D. intortum* + 50% BSFL and T3 = 75% *D. intortum* + 25% BSFL and Control = commercial feed.

## Data Availability

The data presented in this study are available upon request from the corresponding author.

## Supplementary Materials

The following supporting information can be downloaded at: https://www.mdpi.com/article/10.3390/microorganisms10071351/s1, Figure S1: Cumulative relative composition of bacteria Operational Taxonomic Units at family level observed across the different dietary treatments. The top 30 families with high abundance were selected.
