# Supplementary material for: Insights into the Gut Microbial Communities of Broiler Chicken Fed Black Soldier Fly Larvae-Desmodium-Based Meal as a Dietary Protein Source
Source: Microorganisms. 2022 Jul 5;10(7):1351. doi: 10.3390/microorganisms10071351 (PMC9319420; doi:10.3390/microorganisms10071351)
Supplement: Supplementary file 1 [file microorganisms-10-01351-s001.zip › microorganisms-1797320-supplementary.pdf]

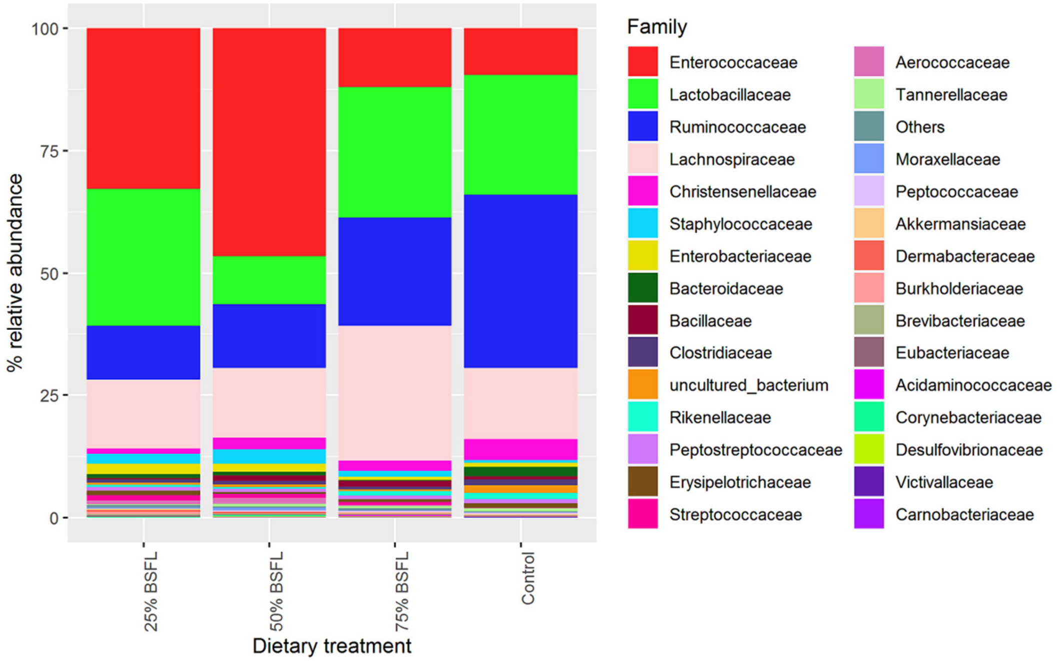

Figure S1: Cumulative relative composition of bacteria Operational Taxonomic Units at family level observed across the different dietary treatments. The top 30 families with high abundance were selected.

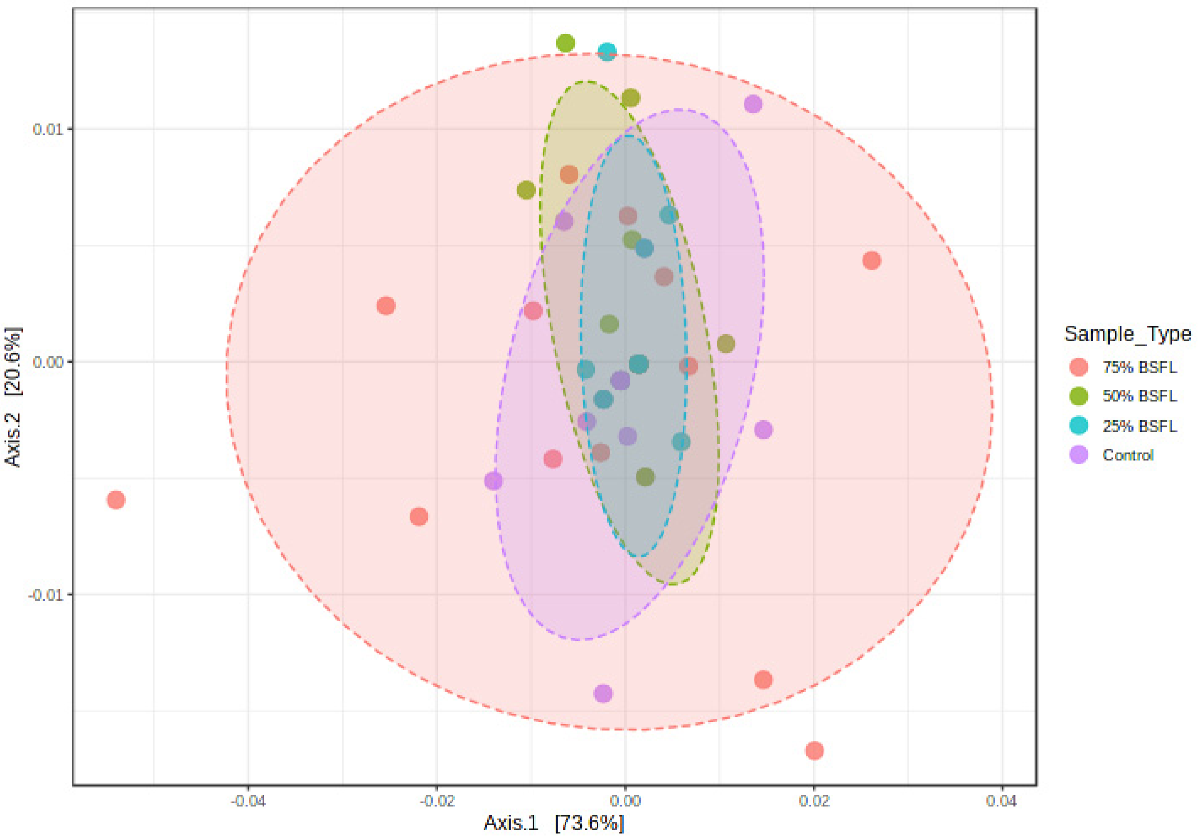

Figure S2: Beta diversity PCoA plot based on Unifrac distance between the different dietary treatments. [PERMANOVA] R-Squared: 0.049; P-value > 0.001
